# On the regional variability of averaged cell area estimates for the human corneal endothelium in relation to the extent of polymegethism

**DOI:** 10.1007/s10792-017-0765-2

**Published:** 2017-11-07

**Authors:** M. J. Doughty

**Affiliations:** Department of Vision Sciences, Glasgow-Caledonian University, Cowcaddens Rd, Glasgow, G4 OBA UK

**Keywords:** Corneal endothelium, Morphometry, Cell areas, Human, Polymegethism, Non-contact specular microscopy

## Abstract

**Purpose:**

To assess variability in the coefficient of variation (COV) in cell area estimates when using different numbers of cells for endothelial morphometry.

**Methods:**

Using non-contact specular microscopy images of the corneal endothelium, 4 sets of 20 cases were selected that included 200 cells and had overall (global) COV values of less than 30 (group 1), 31–40 (group 2), 41–50 (group 3) and over 50% (group 4). Subjects could be normal, or had ophthalmic disease (such as diabetes), a history of rigid or soft contact lens wear or were assessed after cataract surgery. A step-wise analysis was undertaken, 20 cells at a time, of the variability in cell area estimates when using different numbers of cells for the calculations.

**Results:**

Variability in the average cell area values was higher if only 20–60 cells were used in the calculations and then tended to decrease. The standard deviation values on these average cell area values and the calculated COV showed the same overall trends and were more than twice as large for endothelia with marked polymegethism. Using more than 100 cells/image in markedly polymegethous endothelia only increased the variability in the calculations.

**Conclusions:**

These analyses indicate that substantial region variability in cell area values can be expected in polymegethous endothelia. The analysis further confirm that using only small numbers of cells (e.g. less than 50/image) in such cases is likely to yield far less reliable estimates of COV.

## Introduction

As viewed in vivo by specular microscopy, the corneal endothelium of young healthy adults appears as a mosaic of cells having uniform size and shape [1, 2]. The cell size, as reported in the most endothelial assessments, is assessed as the endothelial cell density (ECD), in cells/mm^2^, and provides a very useful indicator of the status of the endothelial cell layer [3]. However, when even some of this uniformity is reduced, then actual considerations of the variation in cell size (area) has been considered important. A specific term was introduced to describe the non-uniformity (i.e. heterogeneity) to the endothelial mosaic, namely polymegethism. This estimates the increased variation in cell areas, reported as the coefficient of variation or COV [3].

In early studies [4], it was noted that substantial differences in cell area variation could exist and that any estimates of ECD could be very much dependent on the overall (global) COV assigned to an endothelial cell layer (assessed by wide-field specular microscopy). For the COV estimates themselves, a later retrospective analysis of published endothelial images indicated that the reliability of any COV calculations would be predictably less if cell area heterogeneity appeared to be present, i.e. whether images were subjectively considered to be normal (homogeneous) or showing some evidence of heterogeneity (polymegethism) [5]. The analysis was, however, limited by the fact that relatively few images were available for analysis and some included somewhat fewer cells than others, often less than 100/image. As a result it was not possible to systematically assess how much the reliability in COV calculations might be reduced according to the extent (or severity) of the perceived polymegethism.

The essential basis of determining the extent of polymegethism is to measure the areas of the cells and then to calculate the average value of the cell area and the standard deviation (SD) of this average area value. It is this SD value that is then used to estimate the area variability as a standardised variance, generally known as the coefficient of variation (abbreviated as CV or COV) based on SD/average cell area calculation. This relative variance can be presented as a fraction (e.g. 0.5 for a moderately polymegethous endothelium) or (more usually) as a percentage (e.g. 50%). In general terms, an increased COV could result from the presence of even a few rather larger cells or small cells, or a combination of both [6]. Stated another way, from a theoretical perspective it could be that parts of an endothelial image could be largely normal with only small regions (or portions) of the mosaic showing larger or smaller cells. The overall estimates of COV have been reported to be dependent on the number of cells measured and therefore included in the calculations [5, 7].

Small field endothelial images taken from normal corneas of young adults with modern day instruments can be expected to include over 100 cells [8–11], while in evaluation of corneas after surgical interventions it has been noted that a good quality image should contain at least 75 cells [6]. Assessments of published images indicated that measuring this number of cells (i.e. 75/image) should give reasonably reliable data in terms of predicted variability in cell areas [5]. Notwithstanding, a systematic analysis of the regional variability in endothelial cell areas within a single image and the impact this on the reliability of the COV data does not appear to have been undertaken in relation to the overall cell area variability. This is important since some contemporary investigators have stated that they have opted to measure relatively few cells (≤ 30/image) in undertaking endothelial analyses whether these be of normal corneas, comparing image analysis systems, assessments of diseases such as diabetes or following interventions such as cataract surgery [12–18]. With the use of just a few cells, the estimates of COV (for example) could be influenced by regional variability in cell areas, i.e. the COV data generated (and reported on) could have rather smaller or much larger values. If no indication is provided of the number of cells actually measured, then such uncertainty in COV estimates also exists.

The present analyses were undertaken to assess this possible regional variability in COV values for endothelial images with different extents of polymegethism. This was done by considering the overall average area and COV values for endothelial images and then systematically investigating the effect of using different numbers of cells to actually calculate the COV values.

## Methods

### Subjects

The study was approved by the university-based ethics committee and formed part of ongoing studies on the corneal endothelium of students, staff and patients presenting for routine eye examinations at the eye clinic. Protocols conformed to the Declaration of Helsinki and all subjects provided informed consent, and could be any age over 18 years and be considered as healthy and having normal corneas, or had abnormal corneas because of known ophthalmic disease (such as diabetes), a history of rigid or soft contact lens wear or were assessed after cataract surgery.

### Image acquisition

Single images of the central corneal endothelium were taken using non-contact specular microscopy (Topcon SP-3000P model, although a few were taken with the older model SP-2000P) and the images downloaded to a thermal printer (Sony Videographic Printer, model UP-897). A numerical code ID number was affixed to the print which was then scanned at 400 d.p.i. to generate a JPEG image file. From such files collected over a 10 year period (2007–2016), examples were selected that contained large numbers of clearly defined contiguous cells but with a different extent of polymegethism from mild to marked (see results). These images were reprinted onto A3-sized white paper, the cell outlines of 200 cells manually marked (see results) and numbered in sequence from the top to the bottom of the image. The marking of the cell–cell borders was undertaken on the very highly magnified (A3) prints and so minimising the chance of any errors, with the author having many years of experience in undertaking this cell marking process. The areas of the outlined cells were then measured by manual planimetry as previously detailed, with this process, especially on the enlarged prints, being expected to be to repeatable (and accurate, as based on the image scale marker) to within ± 2% or better [19, 20].

### Statistical analyses

Using spread sheets in Systat v.11 (Systat, Evanston, IL), the average area values from sequential sets of 20 cells (i.e. numbers 1–20, 21–40 from the top of the images) were calculated. These sets of regional estimates of the average cell areas (from groups of 20 cells) were then used to calculate a progressive estimate of the overall average cell area based on calculating the numerical mean of the average values obtained from 3 regions (60 cells in total), 4 regions (80 cells), etc. up to 10 regions (200 total cells/image). The SD on these estimates was also calculated as well as the COV values (for 3, 4, 5 regions, etc.). Box plots were generated to illustrate the overall variability. All data sets were checked for normality using the default Shapiro–Wilk option in Systat. Where appropriate, 2 sample t tests (for normally distributed data sets) and rank-ordered Friedman tests (nonparametric) were used for comparisons with statistical significance set at *p* < 0.05.

## Results

**Fig. 1:**
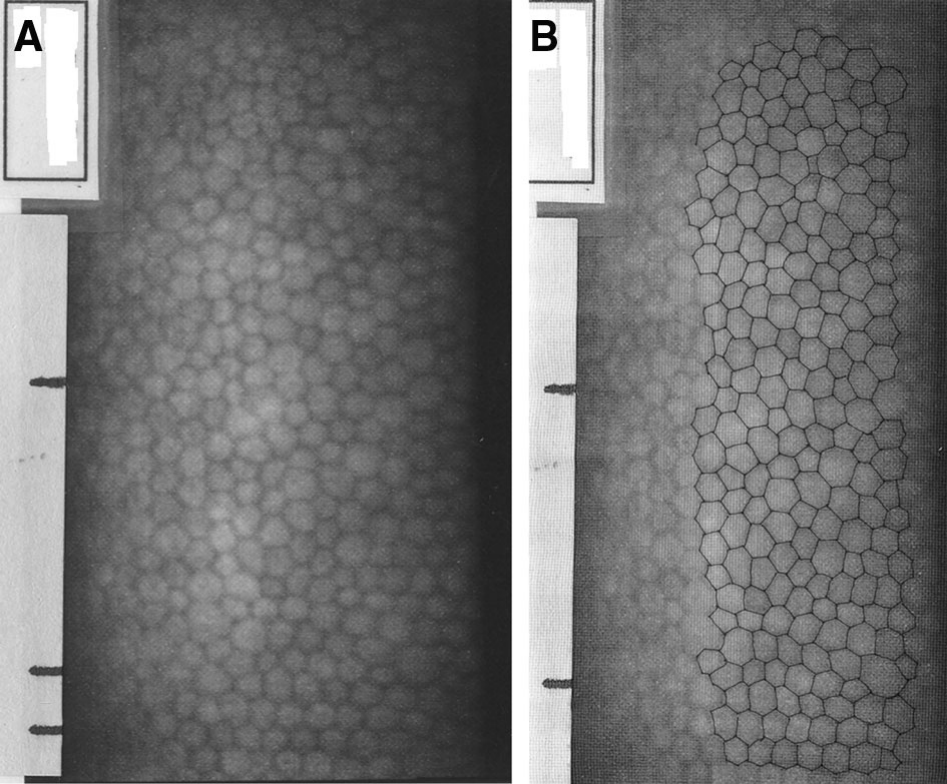
**a** Representative normal endothelium with remarkable homogeneity or lack of polymegethism, **b** same image with 200 cells marked, **c** regions of cells marked on overlay to illustrate only slight regional differences in cell areas

**Fig. 2:**
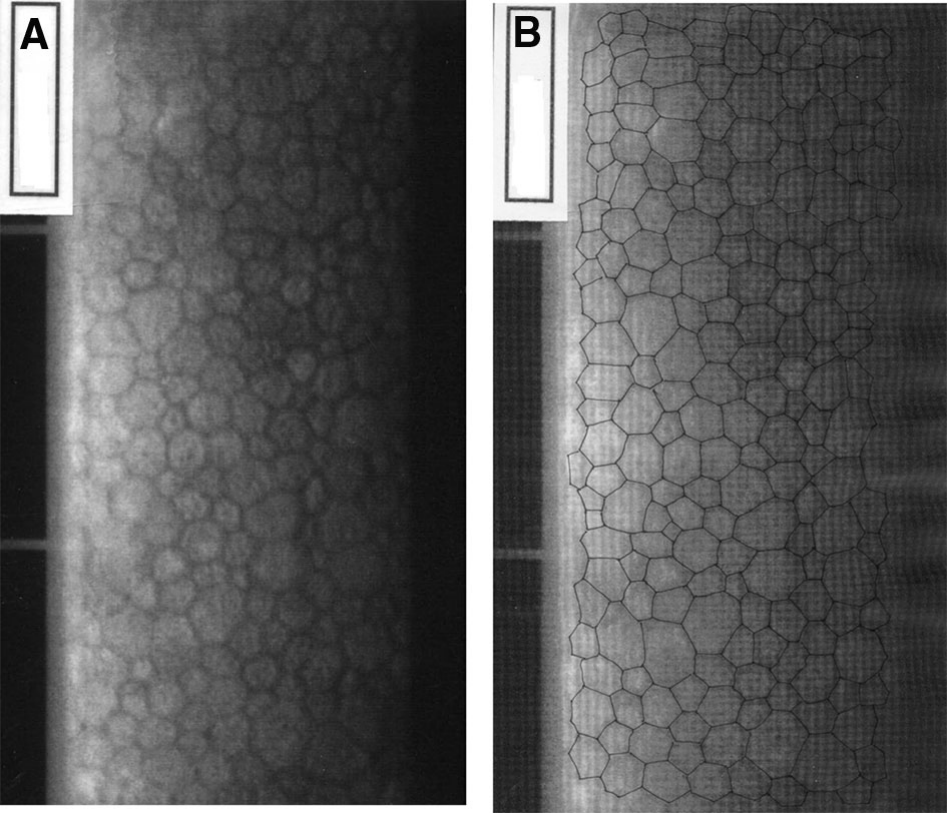
**a** Example of a post-cataract extraction corneal endothelium with notable heterogeneity (moderate polymegethism), **b** same image with 200 cells marked, **c** regions of cells marked on overlay to illustrate remarkable regional differences in cell areas

Two pairs of representative endothelial images used for this study are shown in Figs. 1 and 2 to illustrate the overall image quality and the manual marking of the cell borders. Figure 1 shows a uniform endothelial mosaic with well over 300 cells visible and with most of them having a rather similar size (area). Figure 2 shows a somewhat lower cell density to the image in Fig. 1 and so only includes a little over 200 cells which have a notable range of areas, i.e. show rather marked polymegethism. In the latter image, some of the cells have similar sizes to those seen in the uniform (normal) mosaic but also cells that are distinctly larger or slightly smaller than normal. This regional difference will be specifically addressed later. As noted in the methods, the outlined cells were numbered in sequence from the top of the image to the lower part (not shown). For all 80 endothelial images used in this study, 200 cells were marked and measured. This strategy provided 10 sets of 20 cells for assessment of the regional variation in cell areas in each image.

**Fig. 3:**
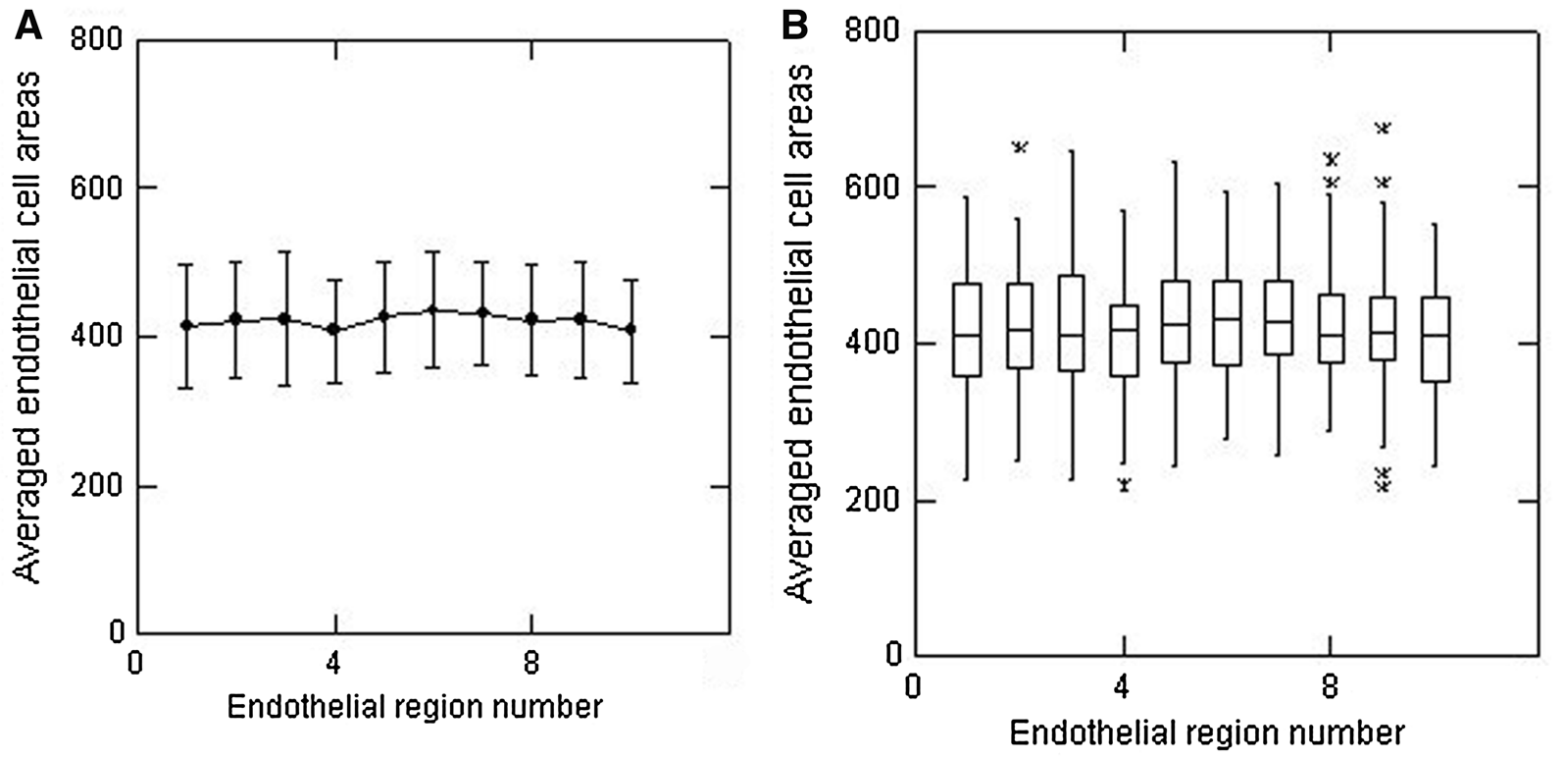
Data from all 80 endothelia to show **a** the group mean (± SD) values for each successive set of 20 cells (region 1, region 2, etc.) from top to bottom of the images, and **b** box plots of the same data to illustrate the median values (horizontal lines), inter-quartile intervals (width of the boxes) and the presence of any outliers (asterisks)

The overall outcome of the analyses of the cell areas within each set of 20 cells for all 80 images is shown in Fig. 3a showing that the group mean values were relatively constant, differing by no more than ± 14 from the overall mean of 421 µm^2^ for all 16,000 cells analysed. Overall, the variability in average cell area values, as assessed by calculating the SD values, was low with most of these values being close to 70 µm^2^. A box plot of the same data shows that the overall data set did, however, include a few outliers, shown as asterisks in Fig. 3b. The box plots also serve to highlight the median area values which, overall, were similar to the calculated group mean values. The sets of values had reasonably constant ± 25% inter-quartile intervals (IQIs), as indicated by the vertical width of the boxes across the plots.

**Fig. 4:**
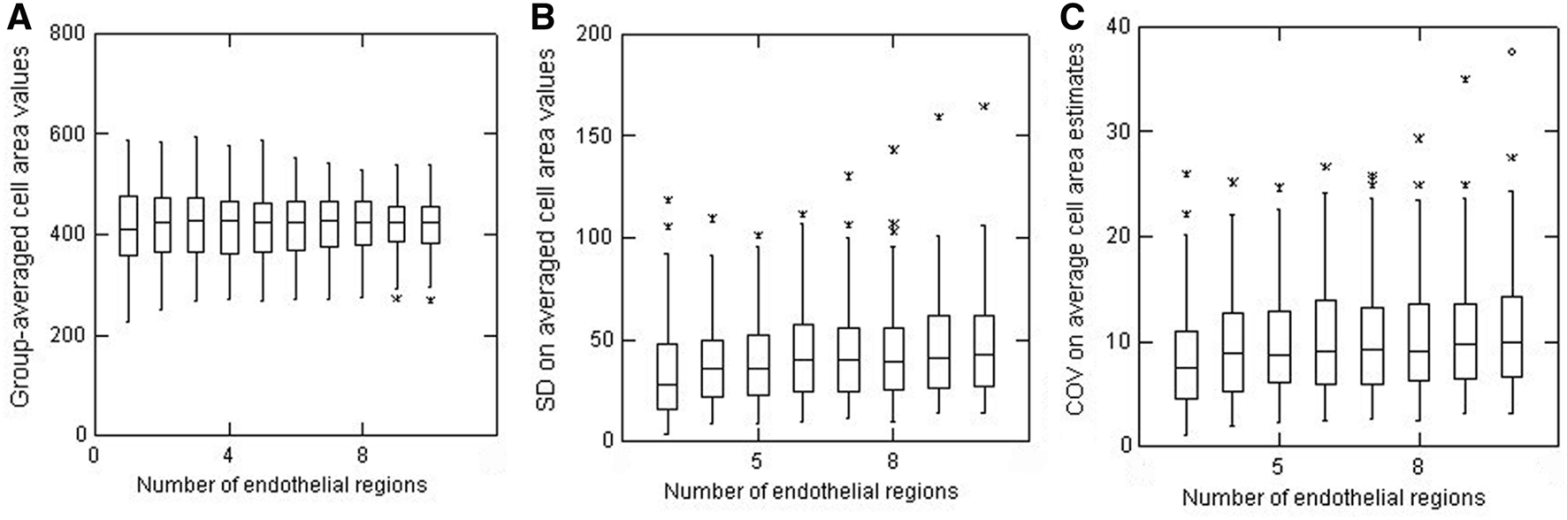
Box plots to illustrate estimates of **a** group-averaged cell area values, **b** the SD on averaged cell area values, and **c** the COV on averaged cell area estimates according to the number of regions of endothelia analysed (from 3 containing 60 cells all the way to 10 regions containing a total of 200 cells). Data from all 80 endothelia

With the overall intent of the present study being to assess the impact of using different numbers of cells to estimate the cell area variability, the data from Fig. 3 are presented in a rather different way in Fig. 4a. Each successive data set is now the result of sequential pooling of data, i.e. using just the first 20 cells from each image generates data for one endothelial region, data from the first 40 cells is two endothelial regions combined, etc. all the way to using all 200 cells/image for 10 endothelial regions. Overall, as more and more cells are included in the calculations, the inter-sample variability (as evidenced from the width of the IQIs) shows a small but clear trend to get smaller. The median values for the group-averaged cell area values were all within ± 5 of the overall values from all 16,000 cells. The SD values on these group-averaged cell area values are shown in Fig. 4b which now highlights a few notable outliers and that there is a slight but progressive increase in the SD values as more and more cells are included in the calculations. Since some of the outliers could simply be the result of substantially different absolute values for average cell area (i.e. as could be found comparing endothelia with a high or much lower cell density), the SD data are presented as its normalised value as the COV (Fig. 4c). As might be expected, the extent of the outliers is lessened but, overall, the net result is not that much different from that shown in Fig. 4b.

**Fig. 5:**
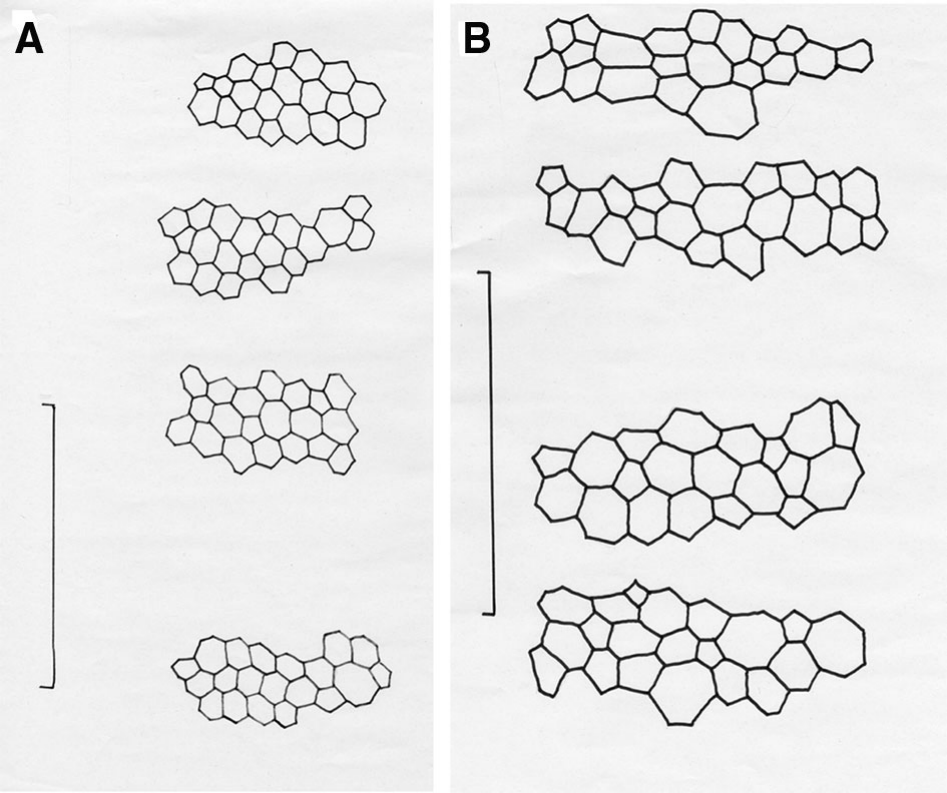
Overlays prepared from marked cells shown in Figs. 1 and 2 to illustrate (**a**) uniform mosaic with on slight differences in cell size in sets of 20 cells, or where there can be marked differences in cell size in sets of 20 cells (**b**)

The analyses just outlined included 16,000 cells divided up into discrete sub-samples of 20 cells as a basis for trying to define uniformity, or identify some non-uniformity, in the endothelial mosaic images. The analysis did reveal a few and sometimes very distinct outliers. That such uniformity can be present is illustrated in Fig. 5a which is an overlay prepared from the image in Fig. 1. For this representative and largely uniform appearing endothelium, the outlined cells are of similar or very similar size (area) in the different regions, but there can still be slight differences that can be seen in the lower right part of the overlay (but not enough to generate outliers in cell size). By notable contrast, as shown in a representative example of moderate-to-marked polymegethism (Fig. 5b), there can be small to substantial differences in cell size even within discrete sets of 20 cells across a single endothelial image.

The main interest in the present study was now to systematically evaluate how such regional differences in average cell area values (for sets of 20 cells) might differ according to the overall extent of the polymegethism considered to be present. For all analyses, the same step-wise progressive averaging was used, i.e. 3 regions averaged (for the first 20 + 20 + 20 cells in each image), regions 1 to 4 averaged (i.e. a total of 4 for the number of endothelial regions used in the calculations), all the way up to 10 regions analysed (200 cells/endothelium). The selected data included four sets of 20 different images which either showed no polymegethism (overall global COV calculation of < 30%) or had different extents of polymegethism from mild (overall global COV of 31–40%), to moderate (overall global COV of 41–50%) to marked (> 50% COV).

**Fig. 6:**
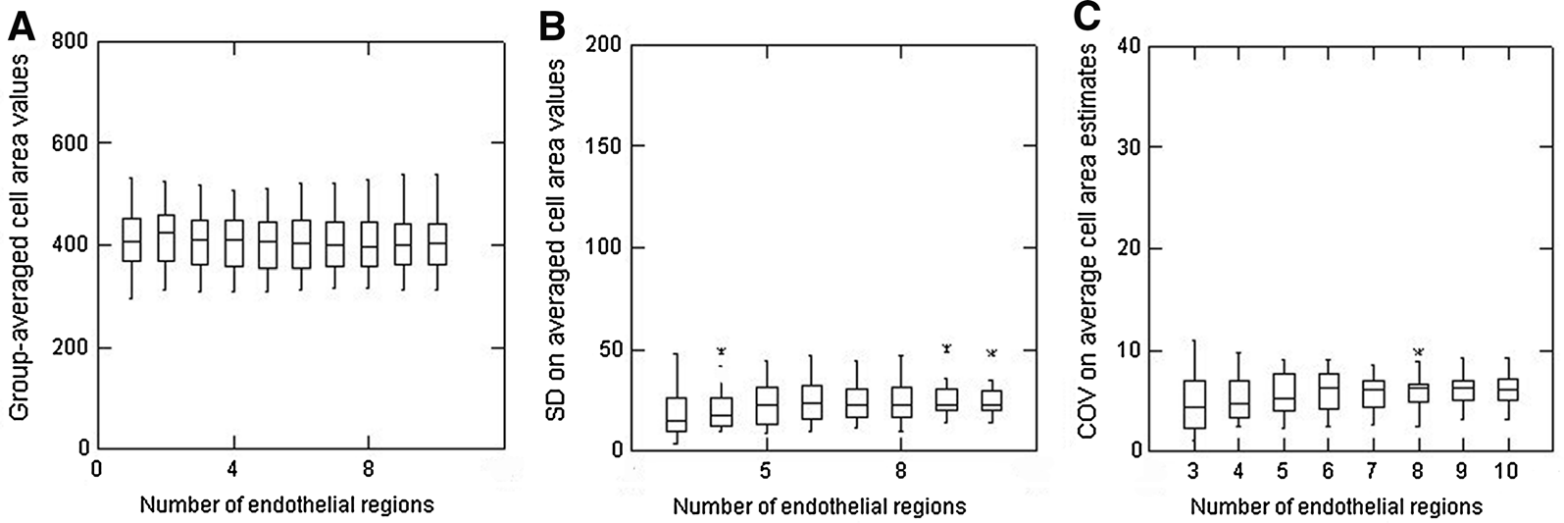
Box plots to show group-averaged cell area values (**a**), the standard deviation (SD) on averaged cell area values (**b**) and the normalised COV on average cell area values (**c**) in relation to the number of regions of endothelia (each including 20 cells) analysed in 20 normal uniform endothelia

The data from 20 endothelia considered to be uniform are shown in Fig. 6. Using different numbers of endothelial regions for the calculations (3, 4, 5, etc. each containing 20 cells), the group-averaged cell area values obtained can be seen to fluctuate slightly but the IQIs were consistent (Fig. 6a). Specific analysis to examine the variability in the group-averaged area values (Fig. 6b) indicates (as expected) that there was a slight trend for the median variability (as SD) to decline as more and more cells were analysed (although the effect was not statistically significant, *p* ≥ 0.1); just 3 outliers were revealed. Overall, a similar result was obtained for assessments of the group-averaged COV for these uniform endothelia with these values and their variability (as indicated by the IQIs) declining slightly when more than 6 regions (i.e. 120 cells)/image were used in the calculations (*p* ≤ 0.05). It should be noted that slightly greater variability in these group-averaged COV values were found if only 3 regions (60 cells), 4 regions (80 cells) or 5 regions (100 cells) were assessed/endothelial image (*p* ≤ 0.05).

**Fig. 7:**
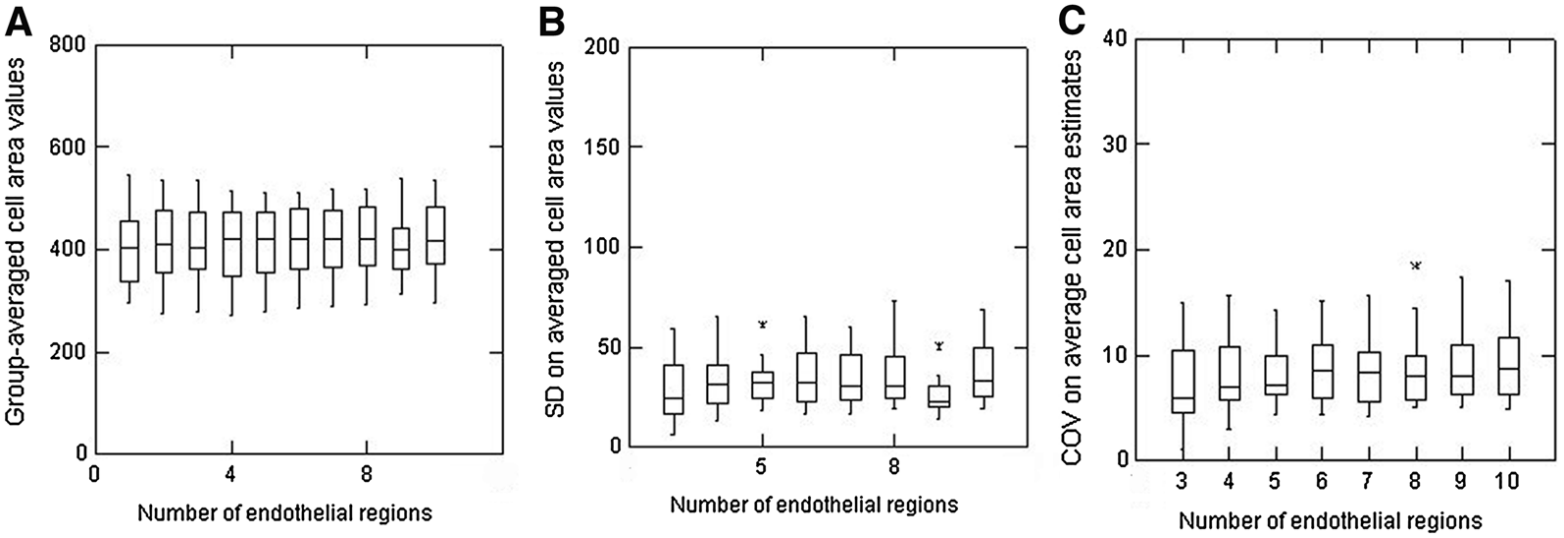
Box plots to show group-averaged cell area values (**a**), the standard deviation (SD) on averaged cell area values (**b**) and the normalised COV on average cell area values (**c**) in relation to the number of regions of endothelia (each including 20 cells) analysed in 20 endothelia considered to show mild polymegethism

The same process was repeated for a set of 20 endothelia with mild polymegethism (Fig. 7) showing group-averaged cell area values to be fairly constant (Fig. 7a), but with slightly greater IQIs than seen for the uniform endothelia. Analysis of the variability in these area values (as the SD) reveals slight inconsistency, however, in that the median values (as well as the IQIs) show quite notable differences when comparing the outcomes from using different numbers of cells (Fig. 7b); two outliers were identified. Analyses of these mildly polymegethous endothelia again indicated greater variability when only 3 (60 cells/image) or 4 regions (80 cells/image) were all that were analysed (*p* ≤ 0.05). Overall, the variability in these group-averaged COV values were slightly greater than for uniform endothelial as indicated by the width of the IQIs and the ± 1.5 SD values (vertical lines above and below the boxes).

**Fig. 8:**
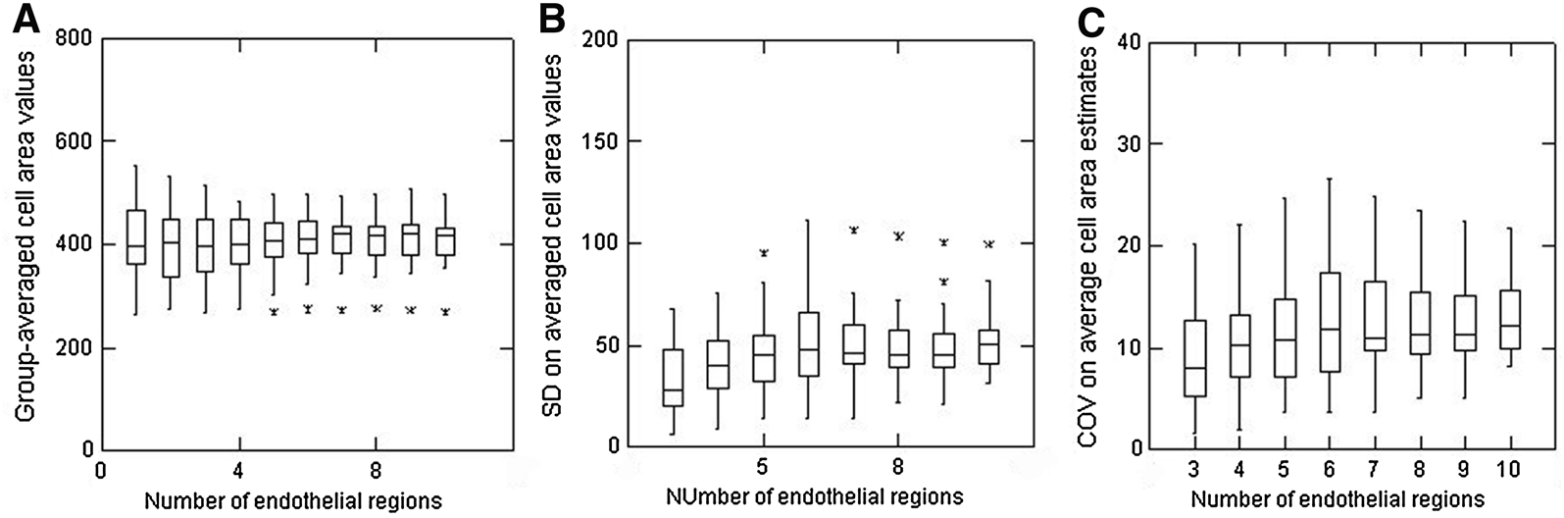
Box plots to show group-averaged cell area values (**a**), the standard deviation (SD) on averaged cell area values (**b**) and the normalised COV on average cell area values (**c**) in relation to the number of regions of endothelia (each including 20 cells) analysed in 20 endothelia considered to show moderate polymegethism

For endothelia showing moderate polymegethism, analysis of the group-averaged cell areas reveals a set of outliers, but the overall trend was for the consistency of these calculations to get progressively better as more and more cells were included in the calculations (Fig. 8a). The notably larger IQIs when only 3, 4 or 5 endothelial regions were analysed should be noted (a result that was again statistically significant, *p* ≤ 0.05). Overall, analyses of endothelia with moderate polymegethism according to the numbers of cells analysed showed obvious variability in SD values for the group-averaged ell areas (Fig. 8b) and a substantial increase in both the overall group-averaged COV values and their variability (as indicated by the notably larger IQIs, Fig. 8c). As with analyses in Figs. 6 and 7, greater COV values and inter-sample variability were noted when 3, 4, 5 and even 6 regions (i.e. 60–120 cells/image) were considered (*p* ≤ 0.05).

**Fig. 9:**
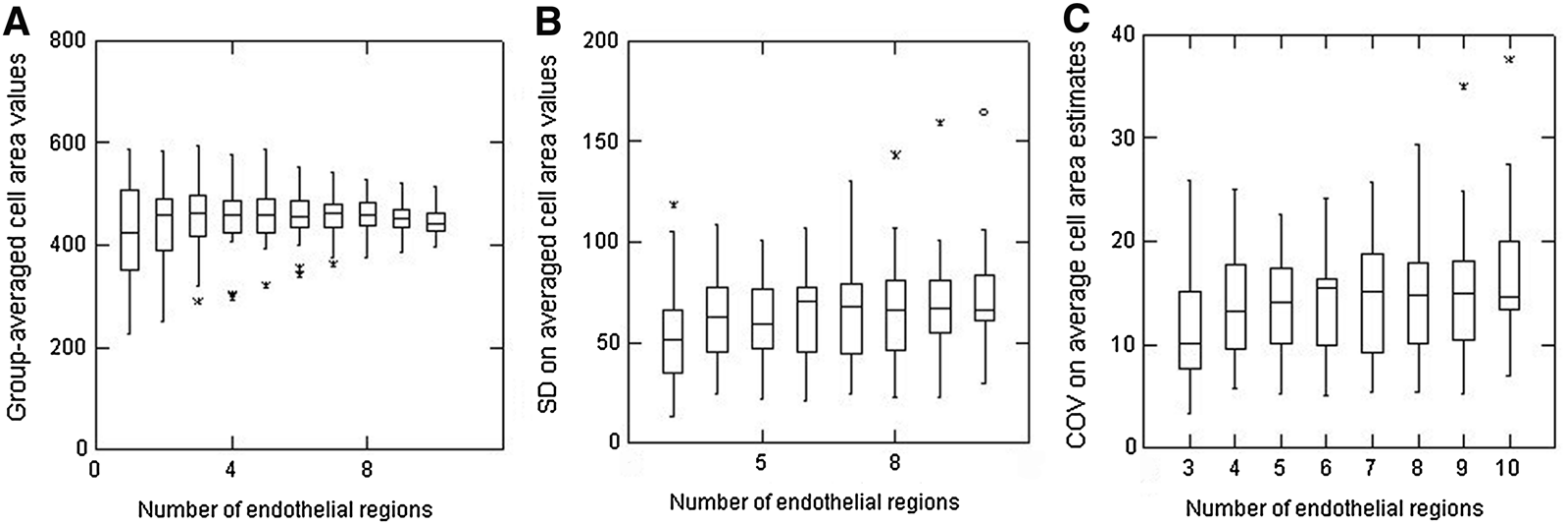
Box plots to show group-averaged cell area values (**a**), the standard deviation (SD) on averaged cell area values (**b**) and the normalised COV on average cell area values (**c**) in relation to the number of regions of endothelia (each including 20 cells) analysed in 20 endothelia considered to show marked polymegethism

The types of effects seen for moderate polymegethism were also seen, but to a slightly greater extent, in 20 endothelia considered to show marked polymegethism (Fig. 9). Of particular note is that the group-average cell area value estimates were more variable when only 60 or 80 cells (3 or 4 regions/image) were analysed [Fig. 9a; (*p* ≤ 0.05)] but then get notably better as more and more cells were included in the calculations. There was no obvious predictability to the SD values on the averaged cell area values (Fig. 9b) and a few notable outliers were identified. Overall, all group-averaged COV estimates were substantially greater (as compared to less polymegethous endothelia; compare IQIs in Figs. 9c and 8c) with the analyses indicting two very notable outliers (with values of > 30% for these internally standardised calculated values).

## Discussion

This study represents the most detailed analysis so far reported of the potential variability in the outcome of corneal endothelial morphometry. It is accepted that these analyses might be considered as a statistical ‘overkill’ in that, essentially, averages of averages from repeated calculations are being generated. The analysis is also presented to illustrate how misleading some global statistics for cell area and COV values can also be. Notwithstanding, the approach used serves the purpose of highlighting cell area variability not revealed in simple global calculations. As illustrated, such group mean values ± SD for cell area values have only limited utility to illustrate any differences. Similarly, the outcome in Fig. 4c again illustrates that the presence of a few outliers can be revealed using box plots (and/or calculations of the IQRs and proportion of outliers) even if other analyses such as those shown in Fig. 4a do not reveal their presence. In reporting comparisons between sets of endothelia, the utility of box plots in revealing important heterogeneity should be noted (and indeed recommended).

These analyses are presented in detail to illustrate that, overall, predictably less reliable results can be expected from corneal endothelial morphometry when less than 100 cells/image are analysed. The results can be applied to most studies undertaken where the overall endothelial cell density is greater than 2000/mm^2^. It is accepted that in some scenarios, it is simply not possible to measure more than 25 cells/small field endothelial image when the endothelial cell density is extremely low (i.e. less 1000/mm^2^) as a result of substantial cell loss in some corneal grafts for example [7]. However, based on published studies over many years, the extent of cell loss following routine cataract surgery (or similar) can now be expected to be very substantially less, and corneas considered suitable for use in graft operations can also be expected to have cell density values above 2000/mm^2^. Therefore, reasonable quality images from post-surgical endothelia should contain 75–100 cells for analyses [7, 21]. Further studies are, however, needed on post-graft endothelia where the cell density values may be considerably lower.

Overall, these analyses are presented to illustrate how any estimates of the variability in cell areas in corneal endothelial images will be expected to be notably different according to the extent of the polymegethism considered to be evident. For this study, a balanced set of examples showing mild, moderate or marked polymegethism were selected for analyses. Overall, a predictable effect is present, illustrated in part C of Figs. 6, 7, 8, 9; as the grade (or extent) of polymegethism increases so also the uncertainty in the estimates of the cell area variability gets greater and greater. It should be noted that these results (part C of Figs. 6, 7, 8, 9) are all normalised to their individual absolute values to avoid any substantial influence of absolute values of the cell areas. The COV estimates given in these figures are not the same as those obtained in overall (global) calculations using all area values from cells from each image. These latter values were essentially up to 30% for uniform endothelia, up to 40% for mild polymegethism, up to 50% for moderate polymegethism and to 60% for marked polymegethism. Based on observations made over many years, these global estimates of COV estimates are considered to be representative of what might be encountered for the types of cases included. Stated another way, a global COV value of less than 20% for human corneal endothelia should be considered as remarkable, and if less than 10% should be carefully scrutinised as possibly an error. The same applies for global COV values in excess of 60% (again for human corneas) as being unusual and possibly an error. Similarly, if the global COV estimates for endothelia considered to be from healthy corneas are 40% or more, then the fidelity of the image analysis should be considered.

The present analyses using these relative values for COV (variability) are presented to try to further illustrate that measuring only a few cells (≤ 50) in small field endothelial images of corneas exhibiting some degree of polymegethism is unlikely to yield acceptably ‘reliable’ estimates for cell morphometry indices. If moderate-to-marked polymegethism is present and only 50 cells or less are used/image then the outcome of any resultant global calculations of COV are unlikely to be reliable, i.e. they could easily be different by ± 10% (or more) with just a few more or a few less cells measured. Such differences could have a substantial impact in deciding the outcome of any comparative studies, especially as to whether or not any statistical differences were detectable (or not). It is hopefully self-evident that it should be incumbent on investigators to provide some reasonable indication of the number of cells measured/image in presenting endothelial analyses. This number, as far as possible, should be consistent when group-by-group comparisons are being made.

As a closing point, the present analyses show that there does not appear to be any obvious benefit in trying to measure more cells/image when even slight polymegethism is evident. This is because the apparent ‘error’ in the COV estimates does not predictably decrease as the number of measured cells increase. As indicated by McCarey and colleagues [7], a reasonable quality image should contain at least 75 contiguous cells suitable for analysis and so a target count of 75–100 cells/image should be striven for. If, for whatever reason, this is not achievable, then discussion of the results should take into account the added uncertainty in the data obtained. This will apply especially when comparisons are being made between different disease conditions or surgical interventions.
